# Adherence to cardiovascular pharmacotherapy by patients in Iraq: A mixed methods assessment using quantitative dried blood spot analysis and the 8-item Morisky Medication Adherence Scale

**DOI:** 10.1371/journal.pone.0251115

**Published:** 2021-05-14

**Authors:** Ahmed Alalaqi, Graham Lawson, Yaseen Obaid, Sangeeta Tanna

**Affiliations:** 1 Leicester School of Pharmacy, Faculty of Health and Life Sciences, De Montfort University, Leicester, United Kingdom; 2 University of Misan, College of Medicine, Misan, Amara, Iraq; Centre for Cellular and Molecular Biology, INDIA

## Abstract

This study evaluated the adherence to prescribed cardiovascular therapy medications among cardiovascular disease patients attending clinics in Misan, Amara, Iraq. Mixed methods were used to assess medication adherence comprising the Arabic version of the eight-item Morisky Medication Adherence Scale (MMAS-8) and determination of drug concentrations in patient dried blood spot (DBS) samples by liquid chromatography-high resolution mass spectrometry. Three hundred and three Iraqi patients (median age 53 years, 50.5% female) who had been taking one or more of the nine commonly prescribed cardiovascular medications (amlodipine, atenolol, atorvastatin, bisoprolol, diltiazem, lisinopril, losartan, simvastatin and valsartan) for at least six months were enrolled. For each patient MMAS-8 scores were determined alongside drug concentrations in their dried blood spot samples. Results from the standardized questionnaire showed that adherence was 81.8% in comparison with 50.8% obtained using the laboratory-based microsample analysis. The agreement between the indirect (MMAS-8) and direct (DBS analysis) assessment approaches to assessing medication adherence showed significantly poor agreement (kappa = 0.28, P = 0.001). The indirect and direct assessment approaches showed no significant correlation between nonadherence to prescribed cardiovascular pharmacotherapy and age and gender, but were significantly associated with the number of medications in the patient’s treatment regimen (MMAS-8: Odds Ratio (OR) 1.947, 95% CI, P = 0.001; DBS analysis: OR 2.164, 95% CI, P = 0.001). The MMAS-8 results highlighted reasons for nonadherence to prescribed cardiovascular pharmacotherapy in this patient population whilst the objective DBS analysis approach gave valuable information about nonadherence to each medication in the patient’s treatment regimen. DBS sampling, due its minimally invasive nature, convenience and ease of transport is a useful alternative matrix to monitor adherence objectively in Iraq to cardiovascular pharmacotherapy. This information combined with MMAS-8 can provide clinicians with an evidence-based novel approach to implement intervention strategies to optimise and personalise cardiovascular pharmacotherapy in the Iraqi population and thereby improve patient health outcomes.

## Introduction

Cardiovascular diseases (CVD) cover disorders of the blood vessels and heart and include hypertension, angina, heart attack, and stroke. Globally, CVD are a major cause of death, accounting for 17.9 million deaths per year in 2016 representing 31% of the total deaths worldwide [1]. In Iraq, non-communicable diseases, including CVD constitute a rising disease burden after the 2003 war [2] and according to the World Health Organisation account for 27% of all deaths in Iraq in 2016 [3].

Cardiovascular disease patients require lifelong treatment with a combination of medications including hypolipidemic drugs, antihypertensives, antiplatelet drugs, diuretics and anticoagulants. The most commonly prescribed CVD drugs in Iraq are amlodipine, atenolol, atorvastatin, bisoprolol, diltiazem, losartan, simvastatin and valsartan [4]. A vital component of managing CVD properly and ensuring treatment success is to ensure patients take their medication(s) as prescribed. The drug selected and the dose prescribed should produce therapeutic drug levels in the patient’s bloodstream. Patient adherence to the prescribed pharmacotherapy helps ensure that the blood drug concentration is within the therapeutic limits in order to improve clinical outcomes. Nonadherence to medications is documented to be a major issue in situations where self-administration of oral medications is required [5] and in situations where polypharmacy exists [6, 7]. There is evidence that as many as 50% of prescribed CVD medications are not taken by patients as recommended [8–10]. This suboptimal adherence to prescribed CVD therapies can lead to substantial health consequences for patients and negative consequences for national healthcare systems because nonadherence reduces the effectiveness of the drug treatment and is associated with morbidity, mortality, medicines wastage, hospital admissions and higher costs of care [9, 11–13]. In USA, nonadherence to prescribed drug therapy is the cause of approximately 125,000 avoidable deaths annually and accounts for $100-$300 billion annually in avoidable healthcare costs to their healthcare systems [5]. Medication adherence is a multifaceted problem that can be influenced by the interplay of patient, treatment and healthcare system-related factors [5]. In lower income countries and middle-income countries, such as Iraq, the prevalence of substandard and falsified medicines may exacerbate this healthcare problem by giving rise to unintentional nonadherence [5, 14]. Given the high humanistic and economic cost associated with nonadherence to prescribed CVD pharmacotherapies the assessment of adherence is a crucial step to ensure that clinicians make an informed clinical decision about treatment and that patients derive the full benefits of the prescribed pharmacotherapy and reduce medicines wastage and costs for healthcare services.

Currently, there is no “gold standard” method for assessing medication adherence in routine clinical practice but a multitude of methods have been employed since each method has its advantages and limitations [5, 15]. These include indirect methods such as pill counts, questionnaires, patient diaries or self-reports, and prescription refill rates as well as direct methods such as measurement of blood or urine drug or metabolite levels [16]. Indirect methods are easy to use and low cost but cannot confirm if the patient has taken their medication correctly and are proxy measures of medication adherence [16]. For instance, pill counts simply confirm the number of tablets removed from their original container but cannot confirm if these tablets have been consumed by the patient. Furthermore, this assessment method provides no information about the time a dose was taken which may be crucial in establishing clinical outcomes [5]. One of the most commonly used standardized questionnaires to assess adherence to cardiovascular therapy medications is the 8-item Morisky Medication Adherence Scale (MMAS-8) [17–20]. Although widely used this self-administered validated tool can be subject to overestimation, recall bias and is unable to assess adherence to each medication in a patient’s treatment regimen. Furthermore, it is unable to take into consideration drug pharmacokinetic and pharmacodynamic parameters which vary from patient to patient. Direct assessment approaches are more accurate methods of measuring medication adherence, however, current measures are costlier in terms of both patient and clinician time and acquiring liquid blood samples requires a visit to a clinic or hospital. The collection of urine samples is non-invasive; however, some patient groups may be reluctant to provide this biosample due to religious, cultural or ethical issues [21]. Tanna and Lawson postulate that the costs associated with direct assessment can be reduced without detriment to the information produced, by use of a fingerpick blood sample collected as a microsample such as a dried blood spot (DBS) for the determination of drug as a measure of medication adherence [21–23]. The ease of sample collection, storage and transport coupled with presenting a low biohazard risk offered by microsampling methods such as DBS mean that it is a viable option to objectively assess medication adherence especially in resource-limited settings [21, 24, 25]. Furthermore, it eliminates the need of phlebotomy thereby facilitating self or remote collection of the DBS [26]. In Iraq, only questionnaire-based assessment of adherence to antihypertensives has been conducted to date and have shown nonadherence to these drug therapies to be prevalent [27, 28].

The aim of this study was to assess Iraqi patients’ adherence to treatment with atenolol, amlodipine, atorvastatin, bisoprolol, diltiazem, lisinopril, losartan, simvastatin and valsartan by using MMAS-8 coupled with the determination of these target cardiovascular medications in DBS samples collected from the same patients and to compare the results between these two assessment strategies.

## Materials and methods

### Study design and ethical approval

The study sample size was 303 and the sample size calculation was determined based on the assumption that the prevalence of CVD in Iraq is 27%, which is close to the prevalence of hypertension at 26.5% [29]. Cardiovascular disease prevalence in Iraq is not documented and therefore the prevalence of hypertension was applied in the sample size calculation using the Daniel equation [30]. Ethical approval for this study was obtained from the Ethics Committee in Misan Health Directorate and De Montfort University’s Faculty of Health and Life Science Research Ethics Committee.

### Participants

Participants were recruited from Alsader Teaching Hospital and Misan Cardiac Centre in Misan, Amara, Iraq during a routine clinical visit between July 2016 and March 2018. Eligible participants were patients aged ≥18 years, and able to understand and communicate in Arabic, had no visual or cognitive impairments and who had been prescribed one or more of the target CVD medications for more than six months prior to recruitment. The target CVD medications were amlodipine, atenolol, atorvastatin, bisoprolol, diltiazem, lisinopril, losartan, simvastatin and valsartan. Pregnant women and non-Arabic speakers, and participants that required admission were excluded. The clinician (YO) screened patients during the study period by applying the selection criteria when attending their appointment and assessed for eligibility. If eligible, the patients were invited to participate and directed to talk to the researcher (AA). The researcher then re-checked the eligibility and those who agreed to participate were provided information about the study by the researcher who gave each patient an Arabic participant information leaflet and obtained written informed consent.

For each participant adherence to prescribed cardiovascular therapies was assessed in two ways:

1. MMAS-8 adherence questionnaire
2. Collection of simple finger-prick blood samples and their subsequent analysis at De Montfort University, UK.

### MMAS-8 adherence questionnaire

Medication adherence was assessed using the structured and validated 8-item Morisky questionnaire, MMAS-8. A validated Arabic translated version of MMAS-8 was used in this study since Arabic is the national language in Iraq. This questionnaire consists of eight questions, the first seven items have a dichotomous answer (yes/no) that indicates adherent or nonadherent behavior. The eighth item has a 5-point Likert scale indicating low to high level of adherence. MMAS-8 total scores can range from 0 to 8 points. Using this standardized questionnaire scores equaling 8, 6 to <8, or <6, patients can be categorized as having high, medium or low adherence to pharmacotherapy, respectively. The MMAS-8 adherence was dichotomized where patients were classified as adherent or nonadherent instead of low, medium and high adherence using a cutoff point of score 6 in MMAS-8. The validity of this dichotomization has been previously published by the developer of the questionnaire [31].

### Collection of patient blood spot samples and baseline medication information

Prior to collection of the DBS samples each patient was required to supply baseline medication data on the number and name of prescribed CVD medicines, dose, dose frequency, approximate time since last dose and name of other prescribed medications. This was via a mini CVD drug prescription questionnaire translated in Arabic. This baseline information coupled with CVD drug pharmacokinetic information would allow the researchers to establish if the calculated blood drug concentration was within the therapeutic window and if the patient had been adherent or nonadherent [21].

Participant DBS samples were obtained by a simple fingerprick using a sterile lancet and the drops of blood were collected on a Whatman 903 DBS sample collection card (GE Healthcare). Each DBS sample collection card was then allowed to dry at room temperature for 2–3 hours. After drying each card was stored in an individual labelled plastic re-sealable bag, containing desiccant, and shipped to De Montfort University, UK under ambient conditions for analysis.

### Extraction and analysis of patient DBS samples

Solvent extraction of the target analytes from DBS was carried out using the protocol detailed in our previously published work [32, 33]. The concentrations of amlodipine, atenolol, atorvastatin, bisoprolol, diltiazem, lisinopril, losartan, simvastatin and valsartan in DBS extracts were determined using a previously validated liquid chromatography-high resolution mass spectrometry (LC-HRMS) method on an Agilent 1290 LC coupled to an Agilent G6530A QTOF mass spectrometer [32, 33]. This bioanalytical validation showed that all target analytes were stable in DBS samples for ten weeks at room temperature. All DBS samples collected in Iraq were transported to the UK and analysed well within ten weeks of collection.

### Adherence assessment based on the LC-HRMS analysis of DBS samples

Patients were considered nonadherent by DBS analysis when one or more of their prescribed CVD medication concentrations were non-detectable or < 5.25% of published C_max_ or > published C_max_ [21, 23].

### Statistical analysis

Statistical analyses were conducted using SPSS software (Version 22.0. Armonk, NY: IBM Corp). Qualitative variables such as gender and medications were expressed in terms of median, interquartile range (IQR), frequencies and percentages. A confidence interval of 95% was employed and a P-value of less than 0.05 was considered significant. A Chi-squared test was used to examine the relationship between levels of nonadherence and gender. Logistic regression was used to examine the relationship between the levels of nonadherence as measured by MMAS-8 and DBS analysis and patients’ age, number of medications each patient is prescribed and the number of different medication tablets taken by each patient. The Kappa concordance test was used to measure the degree of agreement between adherence classified by MMAS-8 and DBS analysis. Mean and standard deviation values were used to express the concentrations of the target CVD medications in the DBS samples for individual patients.

## Results and discussion

### Patient characteristics

A total of 303 participants fulfilled the inclusion criteria of which 49.5% were male and 50.5% female. The median age of the patients was 53years ranging between 30 and 69 years and the median number of medications per regimen was 3 (Table 1). Data on the CVD medications prescribed to patients are presented in Table 2. In this sample 31.1% of the patients were prescribed β-blockers, 25.5% angiotensin II receptor blockers, 16.7% an ace inhibitor, 15.5% statins and 11.2% calcium channel blockers. Bisoprolol (17.5%) was the most widely prescribed medication followed by lisinopril (16.7%) and valsartan (14.8%). Amlodipine (3.4%) and atorvastatin (4.1%) were the least prescribed medications in this cohort.

**Table 1:** Patient population sample characteristics (N = 303).

Variables	Total number of participants = 303	
	N	%
**Gender**		
Male	150	49.5
Female	153	50.5
**Age**		
30–39	25	8.3
40–49	87	28.7
50–59	100	33
60–69	91	30
Median (IQR) = 53 (65–48)		
Q1 (25%) = 48		
Q2 (50%) = 53		
Q3 (75%) = 65		
**Number of medications**		
1–2	130	42.9
3–4	57	18.8
5–6	53	17.5
˃6	63	20.8
Median (IQR) = 3 (6–2)		
Q1 (25%) = 2		
Q2 (50%) = 3		
Q3 (75%) = 6		

IQR: interquartile range.

**Table 2:** Prescribed CVD medications in the Iraqi cohort.

Medication type	N (%) of patients
**β blockers**	
Atenolol	59 (13.5)
Bisoprolol	77 (17.6)
**ACE inhibitor**	
Lisinopril	73 (16.7)
**Angiotensin II receptor blockers**	
Valsartan	65 (14.8)
Losartan	47 (10.7)
**Statins**	
Atorvastatin	18 (4.1)
Simvastatin	50 (11.4)
**Calcium channel blockers**	
Amlodipine	15 (3.4)
Diltiazem	34 (7.8)
**Total**	438 (100)

### Adherence assessed using MMAS-8 questionnaire

The MMAS-8 adherence questionnaire scores ranged from 0 to 8. Based on their MMAS-8 score, patients from the Iraqi cohort were categorized into three groups as described in the methods section: low adherence (MMAS-8 score < 6), medium adherence (MMAS-8 score 6 to < 8) or high adherence (MMAS-8 score 8). As shown in Table 3 more than half of the participants (54.1%) exhibited high adherence, 27.7% exhibited medium adherence and 18.2% exhibited low adherence. In this study patients were classified as adherent or nonadherent rather than low, medium and high using a score of 6 as the cut-off point [31, 34]. Thus 248 participants (81.8%) were adherent to their prescribed CVD pharmacotherapy of which 125 were male (50.4%) and 123 were female (49.6%). The proportion of nonadherent patients, as determined by MMAS-8, was 55 participants (18.2%) of which 25 were male (45.5%) and 30 females (54.5%). This level of nonadherence was similar to a previous questionnaire-based adherence assessment study conducted in Iraq where the reported level of nonadherence was 19.6% [28]. However, it was significantly lower than another questionnaire-based study conducted in Iraq [27]. Adherence to prescribed drug therapy is a complex multifactorial problem that can be influenced by the interrelationship of various factors including patient factors, treatment factors and healthcare system factors [5]. MMAS-8 is unable to assess adherence to multiple medications in the prescribed pharmacotherapy regimens and the assessment of adherence by MMAS-8 is subject to overestimation because this is dependent on the total score obtained from a given patient’s response to the questions. It is possible that patients overestimated their adherence in the current study to a greater degree than in previous studies where lower levels of adherence were documented using a questionnaire-based assessment.

**Table 3:** Adherence amongst Iraqi CVD patients assessed using MMAS-8 score.

Adherence level (score)	Total study population (N = 303)	
	N	%
**Low adherence (< 6)**	55	18.2
**Medium adherence (6 - < 8)**	84	27.7
**High adherence (= 8)**	164	54.1

There was no significant relationship between the level of nonadherence assessed by MMAS-8 and gender (Chi squared = 0.441, df = 1, P = 0.507) and MMAS-8 and age (Odds Ratio (OR) 0.923, 95% CI, P = 0.484). A significant positive correlation between nonadherence and number of medications prescribed (OR 1.947, 95% CI, P = 0.001) and the number of tablets of different medications taken by each patient (OR 1.436, 95% CI, P = 0.001) was shown. As the number of medications taken by a patient increased, the possibility of nonadherence also increased. In the nonadherent group, the mean number of medications taken by patients was 6.53 ±1.63 in comparison with 3.38 ±2.07 in the adherent group.

Based on participant responses to the MMAS-8 questions, 72.3% of nonadherent participants were intentionally nonadherent to their CVD medication. The main reasons for this were medication-related side effects (93.6%), inconvenience of taking the medication (85.1%), financial cost of medications and patients’ belief. The participants reported reasons for medication-related inconvenience were complexity of the regimen, dose frequencies and patient-clinician discordance. Adherence to prescribed drug therapies may be improved by the use of simplified treatment regimens and by reducing the frequency of administration. Patients may prefer medications that require administration on a once daily basis, prescribing the maximum number of doses possible at one time and thus limiting the frequency at which treatment is required. Swapping medications may cause confusion and is inconvenient, and may result in nonadherence [35]. 70.9% of nonadherent patients gave no reason for their refusal to take their medications. By comparison, 27.3% of participants were unintentionally nonadherent and the main reasons for this were forgetfulness and poor understanding of disease.

### Adherence assessed using drug concentrations determined in patient DBS samples

Adherence to a prescribed drug therapy is indicated by the drug level in the blood being between the published C_max_ concentration and 5.25% of C_max_ i.e. the drug concentration after 5 half-lives, when it is considered to be therapeutically inactive. Conversely, nonadherence is indicated by the absence of the drug in the volunteer’s DBS sample or if the drug level determined is outside its therapeutic window [21, 23]. The assessment of nonadherence by determination of the target drug concentration by LC-HRMS analyses of DBS samples from the same volunteers that completed the MMAS-8 questionnaire showed that 154 (82 male and 72 female) patients (50.8%) were adherent to their prescribed CVD medications and 149 (68 male and 81 female) patients (49.2%) were nonadherent. This level of nonadherence is in line with the 50% figure reported by the WHO for nonadherence to medications for chronic illnesses in developed countries [36]. Table 4 gives the breakdown of adherence and nonadherence to each target CVD drug amongst the study participants using the objective patient DBS analysis data. This revealed that nonadherence to the target CVD medications was not uniform. Previous adherence assessment studies in Iraq used indirect methods only and this is the first study to report the use of a direct and objective method to assess adherence to prescribed CVD pharmacotherapy. Thus, there is no previous data about the level of adherence found by direct methods to compare with in Iraq.

**Table 4:** Adherence and nonadherence amongst the Iraqi CVD patient cohort assessed using drug concentrations in patient DBS samples determined using LC-HRMS analyses.

CVD medication	LOQ (ng/ml)	No. of adherent patients (%)	No. of nonadherent patients (%)		
			Patients with no detectable drug concentration <LOQ	Patients with drug concentration in between the LOQ and 5.25% of C_max_	Patients with drug concentration > C_max_
		• Drug concentration in between 5.25% of C_max_ and < C_max_			
**Amlodipine**	0.5	10(66.7)	5(33.3)	-	-
**Atenolol**	10	46(78.0)	9(15.3)	-	4(6.7)
**Atorvastatin**	0.5	8(44.4)	10(55.6)	-	-
**Bisoprolol**	0.1	57(74.0)	20(26.0)	-	-
**Diltiazem**	0.5	20(58.8)	14(41.2)	-	-
**Lisinopril**	0.1	48(65.8)	25(34.2)	-	-
**Losartan**	5	22(46.8)	25(53.2)	-	-
**Simvastatin**	0.1	24(48.0)	26(52.0)	-	-
**Valsartan**	50	33(50.8)	32(49.2)	-	-

All target analytes in the Iraq collected DBS samples were considered stable since all DBS samples were analysed within the validated ten-week stability period [32, 33]. Furthermore, all target analytes from the Iraq patient DBS samples plus quality control (QC) DBS samples within each analytical run were within 15% of their baseline concentration and therefore considered stable.

Adherence assessed using drug concentrations in the biological microsample revealed that gender (Chi squared = 1.707, df = 1, P = 0.185) and age (OR 0.856, 95% CI, P = 0.321) were not significantly associated with the level of nonadherence to prescribed CVD pharmacotherapy. Analogous to the MMAS-8 evaluation, there was a significant positive correlation between with the number of medications in the patient’s prescribed treatment regimen (OR 2.164, 95% CI, P = 0.001) and the number of different tablets taken by each patient (OR 1.607, 95% CI, P = 0.001).

In Table 4 the number of nonadherent patients were categorized in the following three categories: (i) the number of patients with no detectable drug i.e. with drug concentration below the limit of quantification (LOQ); (ii) the number of patients with drug concentration in between the LOQ and 5.25% of the drug C_max_; (iii) the number of patients with drug concentration >C_max_. Ingestion of a medication and LC-HRMS analysis showing non-detectable drug in a patient’s DBS sample is a strong indicator that the patient is likely to have ingested a substandard and/or falsified medicine and was therefore unintentionally nonadherent [5]. In Table 4 the majority of nonadherent patients are in this category. Drug concentrations in between the LOQ and 5.25% of the drug C_max_ would indicate that the patient may be taking their medication but may be skipping doses. No patients in this study were in this category. Drug concentrations >C_max_ indicate that the incorrect medication dose has been taken and four patients were in this category.

### Comparison of medication adherence assessments: MMAS-8 and dried blood spot analysis

To assess the agreement and disagreement between MMAS-8 and blood microsample analysis approaches, the determination of drug concentration in the DBS microsamples was considered to represent the ‘true’ classification of medication adherence. Thus, 248 participants were classified as adherent by MMAS-8 (score > 6). However, DBS analyses showed that only 146 (58.9%) of these 248 participants were actually adherent since their CVD drug blood concentrations determined were between 5.25% of C_max_ and C_max_, the other 102 participants (41.9%) were deemed nonadherent. This suggests the likely overestimation of medication adherence by MMAS-8 for the 102 participants.

Fifty-five participants were classified as nonadherent by MMAS-8, with 47 (88.5%) of these participants confirmed as being nonadherent according to their DBS drug concentrations. The other eight participants (14.5%) were confirmed as being adherent by DBS analysis. This discrepancy may be explained by the acquiescence bias response where the participants give affirmative answers regardless of the content of the question, and where the chances of this form of bias becoming apparent in self-reported questionnaires is quite high [37]. Affirmative answers in MMAS-8 take a value of zero. Thus, the total score will classify patients as being nonadherent. The eight participants who were classified adherent by the DBS analysis responded “YES” to all MMAS-8 questions and thus scored zero.

The agreement between the two approaches to assessing CVD medication adherence was tested via the kappa test, which showed only slight agreement (kappa = 0.28, P = 0.001). This result is different to those reported in other studies which showed that questionnaires were generally highly concordant with biosample drug level measurements [38, 39]. However, these studies either used statistical analysis, such as the Pearson coefficient, which is not recommended for assessment of agreement between two approaches, or used an arbitrary cut-off point in the kappa test. For clinical studies it is recommended that a kappa of 0.8 should be used as a minimum acceptable value for agreement [40].

As is shown in Table 5, agreement and disagreement between MMAS-8 and DBS analyses for each CVD medication showed high agreement for atenolol and bisoprolol at 88.1% and 87.0% respectively, and high disagreement for simvastatin and atorvastatin at 52.0% and 50.0% respectively. However, the overall agreement for nonadherence to prescribed CVD pharmacotherapy as assessed by the kappa test showed only slight agreement between the two approaches (kappa = 0.28, P = 0.001).

**Table 5:** Agreement and disagreement of nonadherence assessment to prescribed CVD pharmacotherapy between MMAS-8 and DBS analysis.

CVD medication	Agreement between MMAS-8 and DBS analysis (%)	Disagreement between MMAS-8 and DBS analysis (%)
**Amlodipine**	66.7	33.3
**Atenolol**	88.1	11.9
**Atorvastatin**	50.0	50.0
**Bisoprolol**	87.0	13.0
**Diltiazem**	73.5	26.5
**Lisinopril**	67.1	32.9
**Losartan**	55.3	44.7
**Simvastatin**	48.0	52.0
**Valsartan**	67.7	33.0

Both assessment approaches showed no correlation between the level of adherence with respect to gender and age. In the literature, there are conflicting results about the correlation between adherence to CVD medications and gender suggesting that complex behavioral factors and sociological gender-based dynamics are at play [4]. The lack of correlation between age and nonadherence to CVD medications is not in line with the findings of another study [34]. Possible reasons for this difference could include various Iraq-specific factors such as the fact that there are no guidelines for the management of CVD in Iraq; there are no CVD medication counselling centres or free healthcare schemes and there is an absence of social support programmes offering appropriate support in Iraq. Thus, nonadherence to prescribed CVD medications might well be expected to affect all age groups in this study. However, the direct and indirect adherence assessment methods employed in this study both showed a significant positive correlation between the level of nonadherence measured and the number of medications taken.

In line with previous studies [41, 42] these results show that regimen complexity and number of prescribed medications influence adherence to prescribed pharmacotherapy. Logistic regression indicated a significant positive correlation between nonadherence assessed by MMAS-8 and the number of medications prescribed (OR 1.947, 95% CI, P = 0.001). As the number of prescribed medications for a patient increased, the possibility of nonadherence to prescribed drug therapy increased accordingly. In the nonadherent patient group, the mean number of prescribed medications was 6.53 ± 1.63 in comparison with 3.38 ± 2.07 in the adherent group. There was also a significant positive correlation between the medication nonadherence assessed by DBS analysis and the number of medications in the prescribed regimen for a patient (OR 2.164, 95% CI, P = 0.001). The mean (±SD) of medications in the nonadherent patients was 5.46 ± 2.15, compared to 2.50 ± 1.40 in the adherent group. Polypharmacy is common practice to control CVD [6, 7] and to improve mortality and morbidity, however it can expose patients to increased risk of adverse drug reactions and drug-drug interactions [43] and is a known contributor to intentional medication nonadherence [5]. The prevalence of substandard and falsified medications in the markets of developing countries such as Iraq could also account for the unintentional nonadherence to prescribed drug therapy and the differences in levels of nonadherence for the different medications [5, 14]. It is postulated that if a patient ingests such poor-quality medicines their blood drug levels will not reach the required therapeutic levels since such medications contain little no active pharmaceutical ingredient and this can lead to treatment failure [5, 14].

**Fig 1:**
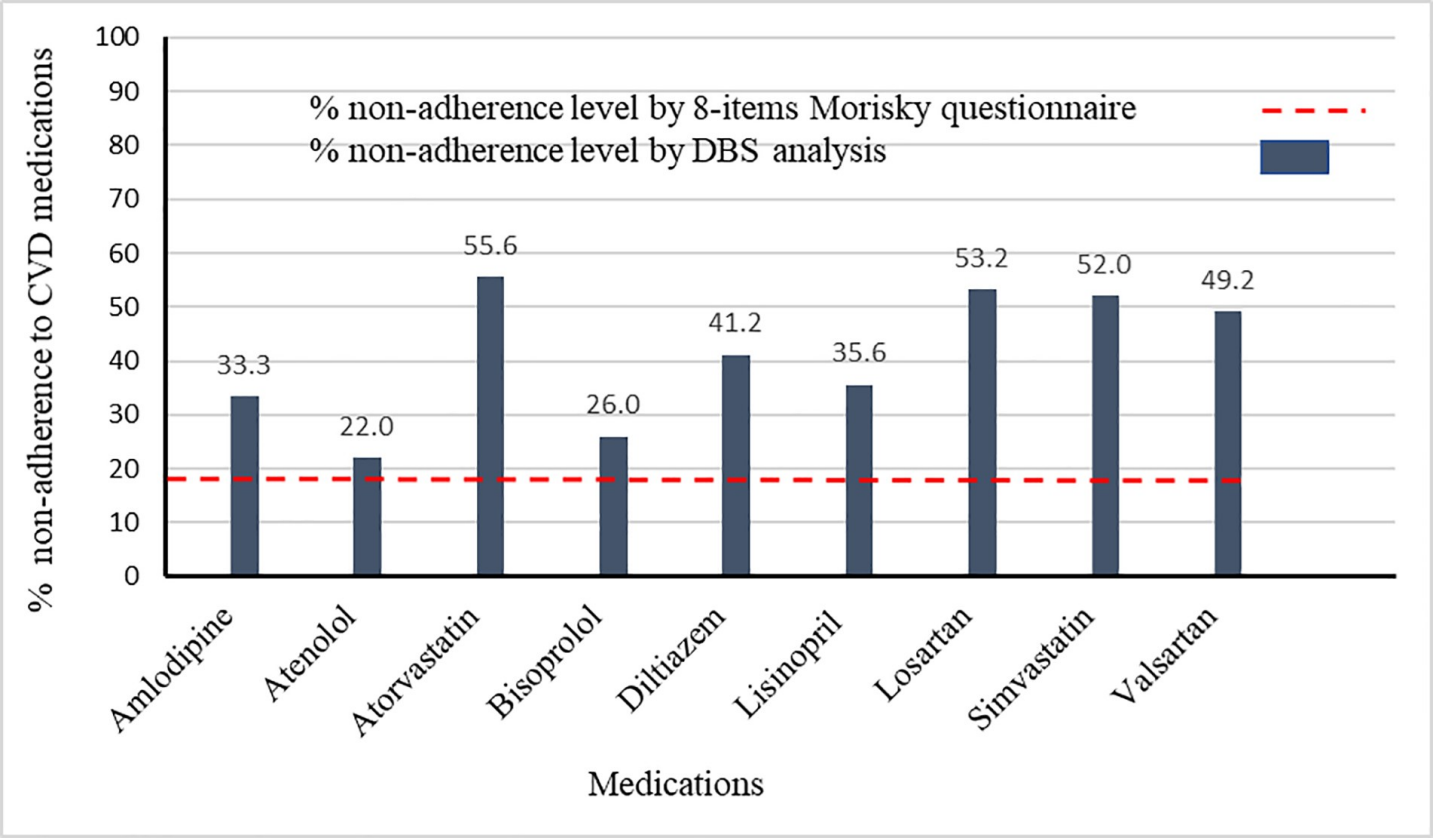
A comparison of nonadherence assessment to prescribed CVD pharmacotherapy between DBS analysis and MMAS-8.

Whilst MMAS-8 is easy to administer to patients it is unable to track nonadherence to each medication in the regimen. Since polypharmacy is common in the treatment of CVD this is a drawback of using this type of indirect assessment method for evaluating adherence to drug therapy. Furthermore, when these indirect methods are employed to assess medication adherence it is not possible to track dosing error and/or prescription error or if the patient took the medication at the wrong time. Patients may take the wrong medication or the incorrect dose or at the wrong time. In these cases, whilst the medication-taking behavior is present the patient will not derive maximum therapeutic benefit from the ingested medication or may experience adverse side effects. Such indirect methods of assessment cannot also take into consideration patient-to-patient variation in pharmacokinetics, pharmacodynamics and pharmacogenetics, which affect drug concentrations in the blood. If a clinician assumes that a patient is taking their prescribed medicine as recommended, he or she may attribute progression of the patient’s CVD condition to a lack of activity of the prescribed CVD drug and therefore may unnecessarily change a regimen [5]. As is evident from Fig 1 the direct assessment method of using the analysis of blood microsamples is able to provide information about the drug concentrations of each drug and thus nonadherence for each medication. This objective information would be helpful to clinicians in terms of optimizing and individualizing each medication in the regimen for each patient. For instance, one patient in this study was prescribed losartan and simvastatin and was categorized as adherent according to his/her MMAS-8 score. However, the LC-HRMS analysis of their DBS sample showed that this patient was only adherent to losartan but not simvastatin and interventions were put in place by the clinician to address this issue. Furthermore, MMAS-8 alone cannot also determine if the patient took the correct dose at the recommended time. For instance, four patients in this study were prescribed atenolol and were categorized as adherent based on MMAS-8, however, three of these patients were deemed nonadherent based on DBS analysis as their blood atenolol concentrations exceeded the C_max_ for the reported dose of atenolol (50 mg and 100 mg). Subsequent patient discussions with the clinician revealed that all three patients had taken more than the prescribed dose in the belief it would lead to improved clinical outcomes. The fourth patient had mistakenly inserted his/her atenolol blister pack in their atorvastatin packaging and so had been taking double the dose of atenolol whilst missing his/her dose of atorvastatin. This explained the high concentration of atenolol and the non-detection of atorvastatin in the DBS sample taken from this patient. In these four situations atenolol tablets were ingested by each of the patients and each patient responded honestly to the MMAS-8 questions but these patients were taking the incorrect dose of atenolol, which MMAS-8 is unable to reveal. The objective assessment of medication adherence using quantitative dried blood spot analysis fits into the framework of personalised medicine and would offer a less costly and more direct approach than genetic analysis for personalised titration of pharmacological interventions. The dual approach of employing indirect and direct methods to measure medication adherence can help healthcare providers to accurately assess adherence and identify barriers associated with nonadherence to prescribed drug therapy. The healthcare providers can then attempt to address the associated problems and inform the patient as to how the problem will be addressed. Triangulation with other methods such as patient interviews could provide additional information.

Fig 1 and Table 4 indicate that nonadherence to CVD medications, in the Iraqi patient cohort, as assessed by DBS analysis is not uniform. Differences in the levels of adherence to prescribed CVD pharmacotherapy can be influenced by the medication class and medication-related side effects. Statins are documented to have lower adherence levels compared to other CVD medications [44]. This study supports these findings where the adherence to atorvastatin and simvastatin was 44.4% and 48.0% respectively versus 78.0% and 74.0% for the two β-blockers, atenolol and bisoprolol respectively. Availability and cost of medications in Iraq could also account for the differences seen in adherence levels for the CVD drugs. In Iraq, atorvastatin, simvastatin and valsartan have a relatively high retail price in comparison with atenolol and bisoprolol. Furthermore, at the time of this study, these statins were only available to patients from the Iraq private healthcare sector. Medication availability in the Iraq public healthcare sector is dependent upon demand of each medication and can change annually. The Iraq private healthcare sector has higher retail prices for all medications compared to the public and intermediate sectors where medication prices are controlled by the Iraq Ministry of Health [4].

## Conclusion

This is the first study to assess adherence to prescribed cardiovascular pharmacotherapy in Iraq via drug concentrations in DBS samples and comparing to MMAS-8 results. This study showed that only 50.8% of the Iraqi volunteers were adherent to one or more of their prescribed CVD medications when assessed using the analysis of patient DBS samples in this patient population. This compared with 81.8% of adherence determined by MMAS-8 suggesting that the assessment of adherence using a standardized questionnaire may be subject to a degree of overestimation. Poor agreement between the two assessment approaches was evident and the DBS derived data provided drug specific information about patient medication taking behavior and revealed that nonadherence was not uniform towards the different medications in the patient drug regimens.

The unique mixed method approach investigated in this study is a very useful approach to adherence assessment in providing an evidence-base to clinicians to make informed clinical decisions for future treatment and intervention(s) development and maximise patient clinical outcomes. The minimally invasive DBS microsample collection method used in this study offers advantages of patient convenience and ease of storage for routine implementation in Iraq. A further benefit of this patient-friendly sample collection method is that the DBS samples can be easily shipped for analyses to laboratories outside Iraq where sophisticated hyphenated mass spectrometry-based systems, required for analyses, are readily available [45]. The direct blood drug concentration method can provide objective information on the levels of each medication in the patient’s blood, thus offering a route for optimization and personalisation of treatment, whilst the indirect standardized questionnaire method highlights possible reasons for nonadherence to prescribed CVD pharmacotherapy. In the event of poor patient progress, it is essential the clinician knows if the patient has followed the prescribed treatment regimen as recommended and clinicians need an evidence-based framework to guide their clinical decisions. The specific medication-related nature of the blood concentration data can aid the clinician to make informed clinical decisions about future treatment and results from the MMAS-8 questionnaire can aid the clinician to initiate evidence-based discussions with the patient to understand exactly the reasons for nonadherence.

The findings of this study also raised another critical healthcare issue for Iraq which is the evidence of the high risk of substandard and/or falsified medicines circulating within the Iraqi healthcare system and unknowingly being made available to patients.

Cardiovascular diseases are a growing healthcare concern in Iraq and the low level of adherence to prescribed CVD pharmacotherapy highlighted in this study requires actions for pharmaceutical care. This study provides the impetus for assessment of medication nonadherence in routine clinical practice in Iraq, using a mixed method approach. This is to maximise patient benefit from the drug therapies prescribed and reduce medicines wastage and healthcare provider costs.
